# Introducing Murine Microbiome Database (MMDB): A Curated Database with Taxonomic Profiling of the Healthy Mouse Gastrointestinal Microbiome

**DOI:** 10.3390/microorganisms7110480

**Published:** 2019-10-23

**Authors:** Junwon Yang, Jonghyun Park, Sein Park, Inwoo Baek, Jongsik Chun

**Affiliations:** 1 Interdisciplinary Program in Bioinformatics, Seoul National University, Seoul 08826, Korea; yjunwon18@gmail.com (J.Y.); warwarsn@naver.com (J.P.); psi103706@gmail.com (S.P.); 2 Institute of Molecular Biology & Genetics, Seoul National University, Seoul 08826, Korea; iwbaek0828@gmail.com; 3 School of Biological Sciences, Seoul National University, Seoul 08826, Korea

**Keywords:** database, metagenomics, mouse gut microbiome

## Abstract

The gut microbiota modulates overall metabolism, the immune system and brain development of the host. The majority of mammalian gut microbiota consists of bacteria. Among various model animals, the mouse has been most widely used in pre-clinical biological experiments. The significant compositional differences in taxonomic profiles among different mouse strains due to gastrointestinal locations, genotypes and vendors have been well documented. However, details of such variations are yet to be elucidated. This study compiled and analyzed 16S rRNA gene-based taxonomic profiles of 554 healthy mouse samples from 14 different projects to construct a comprehensive database of the microbiome of a healthy mouse gastrointestinal tract. The database, named Murine Microbiome Database, should provide researchers with useful taxonomic information and better biological insight about how each taxon, such as genus and species, is associated with locations in the gastrointestinal tract, genotypes and vendors. The database is freely accessible over the Internet.

## 1. Introduction

Mouse, *Mus musculus*, has long been used for biological studies as a mammalian model organism due to its low cost, short generation time and small size [1,2]. In particular, inbred laboratory mouse strains dominate genetic and immunological studies because each strain created by inbreeding over 20 generations is considered genetically identical [3]. The microbiome, also known as the second genome, is the complex community of microorganisms belonging to Archaea, Bacteria, Eukarya and viruses that can be found living both on and in vertebrates and have potential to affect host physiology, immunity and development [4,5,6,7]. Mice have been extensively used in microbiome-related studies which demonstrated a clear correlation between the mouse gut microbiome and host immune response [8,9,10,11,12,13,14,15,16,17,18]. For example, C57BL/6 mice obtained from the Jackson Laboratory and Taconic Biosciences showed substantially different resistances against the melanoma tumor, which resulted from the differences of their gut microbiota [19].

Although inbred laboratory mouse strains, including C57BL/6, have been widely used in biological experiments and pre-clinical testing, it is difficult for most researchers to comprehend the usual taxonomic make-up of mouse gut microbiota and the frequencies of each species or genus in different conditions, such as locations in the gastrointestinal (GI) tract, genotypes or vendors/environments [20,21,22,23,24]. Furthermore, there are no curated databases for the mouse gut microbiome.

In this study, we present the murine microbiome database (MMDB), which contains 16S rRNA gene-based microbiome taxonomic profiles (MTPs) of the healthy mouse gut microbiota with manually curated metadata. By compiling 554 microbiome samples, the overall compositional variations due to locations in the GI tract, genotypes and vendors were unraveled. Additionally, a variety of alpha-diversity indices were calculated to compare species richness and evenness of samples grouped by sampling locations, genotypes or vendors. Also, to assess overall relationships among the samples held in the database, the beta-diversity was calculated and compared using statistical values. Taken together, these results provide further insights into the relationship between the healthy mouse gut microbiome and the various conditions in terms of taxonomic compositions and species diversities within a specific condition or between the different conditions. Furthermore, the database with a web-based user-interface would provide easy access to these findings by the search services, which return the information of interest.

## 2. Materials and Methods

### 2.1. Collection of Metagenome Sequences

Amplicon data based on the bacterial 16S rRNA gene were downloaded from the NCBI Short Read Archive (SRA) database with accompanying metadata. The publications associated with SRA data were manually checked and inserted into the MySQL database (https://www.mysql.com/). Only data labeled as the healthy mice with complete metadata and containing V4 or V3V4 region of 16S rRNA were compiled.

### 2.2. Construction of Microbiome Taxonomic Profile (MTP) Database

Each metagenome sequence was trimmed for V4 region by in-house code to use the same region for further analysis and analyzed using the EzBioCoud pipeline [25] with EzBioCoud 16S database version PKSSU4.0. In the pre-processing step, paired-end reads were merged in the case of paired-end sequencing, and primers used for polymerase chain reaction (PCR) amplification were trimmed by in-house code. Sequences with low quality were filtered out using the following criteria: (i) sequences with the lengths of <100 bp or >2,000 bp, (ii) averaged Q value was <25, (iii) not predicted as a 16S rRNA gene by the hidden Markov model (HMM) based search or (iv) found to be a singleton when all sequences that did not match any of reference sequences with at least 97% similarity cutoff were clustered using the UCLUST program [26] with 97% cutoff. Only non-redundant sequences were extracted and then subjected to the taxonomic assignment using the VSEARCH program [27] to search and calculate the similarity value of the query sequences against the EzBioCloud 16S database using the previously proposed cut-off values [28]. The sequences were checked for chimera using the UCHIME program [29] and operational taxonomic units (OTUs) were picked using the open-reference method [25]. Various alpha diversity indices (ACE, Chao1, Jackknife, Shannon, Simpson, NPShannon, Phylogenetic diversity) were calculated using OTU information. Only samples containing >10,000 valid reads were parsed and inserted into MySQL database after the processes. 

The taxonomic composition of each microbiome sample at all taxonomic ranks was visualized using the Krona tool [30] in the web site. Chao1 and Shannon indices were calculated with the rarefied number of reads (10,175 reads per sample) using the ‘skbio’ package in python (http://scikit-bio.org/docs/0.2.0/generated/skbio.diversity.alpha.html) and visualized using the ‘ggpubr’ package in R [31]. Beta-diversity among the samples was computed with the rarefied number of counts (6266 counts per sample) and visualized by two-dimensional principal coordinates analysis (PCoA) plot of Bray–Curtis distances using the QIIME package [32]. 

### 2.3. Statistics

The Wilcoxon rank-sum test [33] was performed using the ‘ggpubr’ package in R to identify significant differences in the Chao1 and Shannon indices across different sampling locations, genotypes or vendors [31]. The analysis of similarity (ANOSIM) test was employed to see whether the groupings based on given variables for each factor were largely separate and statistically significant using the QIIME2 package [34].

### 2.4. Operating System and Programming Languages

The database and web site were constructed using MySQL, Spring Boot (https://spring.io/projects/spring-boot) and JAVA under the Linux operating system.

## 3. Results and Discussion

### 3.1. Taxonomic Profiles of Heathy Mouse Gastrointestinal Microbiome

A total of 33,948,903 sequencing reads (554 samples from 14 projects) were processed and used to generate taxonomic profile data after filtering by quality and chimera-detection. More than 4700 species and 1700 genera were found in 554 mouse microbiome samples (Table 1). When microbiome samples were considered in 7 locations in the GI tract, interestingly, the most frequently found species were different in all areas (Table 2 and http://leb.snu.ac.kr/mmdb/frequency). The most frequently found bacterial species in feces was phylotype PAC001071_s, a tentative species that is represented by an uncultured sequence (nomenclature according to Yoon et al. [25]). This phylotype was found in 77.13% of samples with at least 1% relative abundance and belonged to the family *Muribaculaceae*, formerly known as the S24-7 group [35]. The members of this family were repeatedly reported as common inhabitants of the mouse gut microbiome [36,37,38,39]. In contrast, uncultured phylotype PAC001188_s belonging to the genus *Oscillibacter* was most frequently found in mouse cecum (66.67% of samples). In mouse duodenum, *Akkermansia muciniphila* that is considered one of the most important human intestinal bacteria, was second most frequently found.

**Figure 1:**
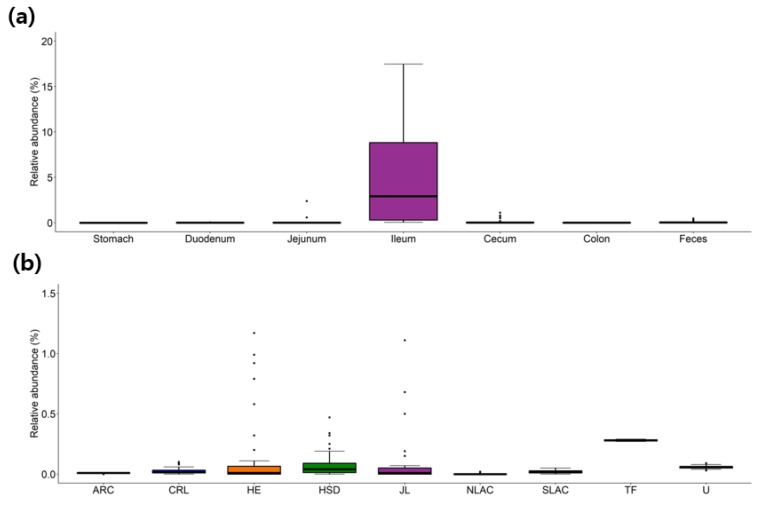
The relative abundance of the *Candidatus* genus Arthromitus (also known as segmented filamentous bacteria, SFB) (**a**) along the mouse gastrointestinal tract or (**b**) from different vendors. Data is accessible at http://leb.snu.ac.kr/mmdb/taxon/Arthromitus. ARC, Animal Resource Centre; CRL, Charles River Laboratories; HE, Harlan Envigo; HSD, Harlan Sprague Dawley; JL, Jackson Laboratory; NLAC, National Laboratory Animal Center; SLAC, Shanghai Laboratory Animal Center; TF, Taconic farms; U, UAMS.

Additionally, a relative abundance of each taxon at different locations in the GI tract was also visualized as a series of boxplots, which provides users straight forward information about the distribution of each species or higher taxa. For example, the overall distribution of strains belonging to the *Candidatus* genus Arthromitus, previous known as segmented filamentous bacteria (SFB), along the GI tract of the healthy mice, is visualized in Figure 1a. The SFB has been extensively studied as an important modulator of mouse immunity [40] and is known to be present in a higher proportion in mice obtained from Taconic Farms than Jackson Laboratory [40], which was confirmed in our database (Figure 1b).

### 3.2. Alpha-Diversity Variations in the Healthy Mice

**Figure 2:**
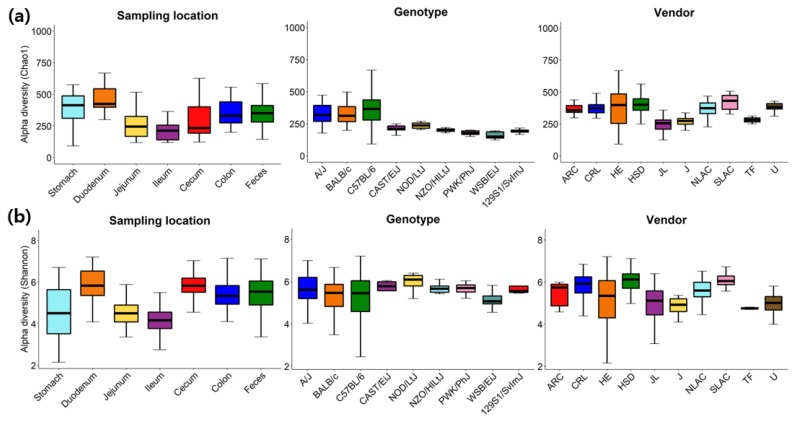
Alpha diversity of samples grouped by sampling locations, genotypes or vendors as measured using (**a**) Chao1 and (**b**) Shannon with the rarefied number of reads (10,175 reads per sample). ARC, Animal Resource Centre; CRL, Charles River Laboratories; HE, Harlan Envigo; HSD, Harlan Sprague Dawley; JL, Jackson Laboratory; J, Janvier; NLAC, National Laboratory Animal Center; SLAC, Shanghai Laboratory Animal Center; TF, Taconic farms; U, UAMS.

A variety of alpha-diversity indices (ACE, Chao1, Jackknife, Shannon, Simpson, NPShannon, Phylogenetic diversity) were calculated and provided for each microbiome sample along with the rarefaction curve at the web site (e.g., see http://leb.snu.ac.kr/mmdb/sample/SRS347361 for a cecum sample of a PWK/PhJ mouse from Jackson Laboratory). The distributions of Chao1 and Shannon were further analyzed with the rarefied number of reads (10,175 reads per sample) in detail (Figure 2 and Table S1). As reported in multiple studies [41,42,43,44], species richness and evenness of microbiome samples from different GI locations, genotypes and vendors varied substantially. Species richness indicated by Chao1 is expected to be higher in the feces than ileum and jejunum [41], and species evenness indicated by Shannon is expected to be higher in the cecum, colon and feces than ileum and jejunum [44] which can be easily confirmed in Figure 2a and Table S1.

### 3.3. Beta-Diversity

**Figure 3:**
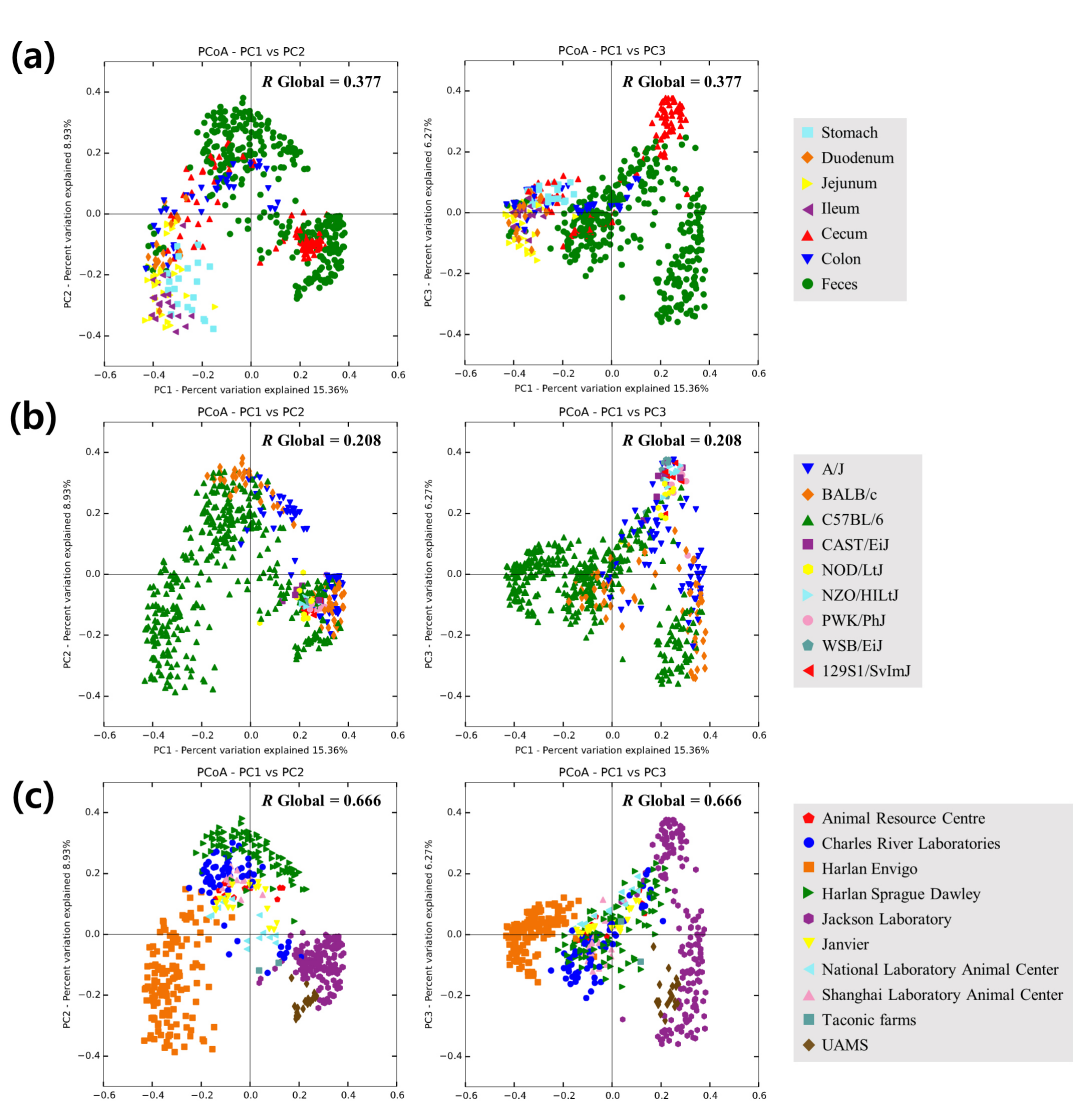
Two-dimensional principal coordinates analysis plot of Bray–Curtis distances with the rarefied number of counts (6266 counts per sample) among the samples. Samples were grouped by (**a**) sampling locations, (**b**) genotypes or (**c**) vendors. Analysis of similarity (ANOSIM) *R* value is indicated in the upper right of each graph (*p* = 0.001 with 999 permutations).

To assess overall relationships among the samples held in the database, the beta-diversity was calculated with the rarefied number of counts (6,266 counts per sample) using Bray–Curtis distances and principal coordinates analysis (PCoA). Bacterial communities in the cecum, colon and feces samples are expected to cluster closely to one another [44], and it was also found in PCoA plot (Figure 3a) and ANOSIM pairwise comparisons (Table S2). The type of vendor (ANOSIM *R* = 0.666 and *p* = 0.001; Figure 3b) was a significant factor for the difference in taxonomic composition, whereas the genotype (ANOSIM *R* = 0.208 and *p* = 0.001; Figure 3c) was a less significant factor for the difference, which was similar result to Ericsson et al. [21].

## 4. Conclusions

This study gathered taxonomic profiles of 554 gastrointestinal tract microbiome samples of healthy mice, then organized the data into an interactively browsable web-interface, named MMDB. As a result, a detail report on the taxonomic profile and alpha-diversity indices of each microbiome sample is provided. In addition, users of MMDB can easily find the distribution of each bacterial taxon, including > 4700 species and > 1700 genera, along with the locations in the GI tract or differences in genotypes or vendors, or combination of them. Taken together, it is fair to say that users of MMDB can easily access the information of healthy mouse gastrointestinal microbiome with curated metadata, and the information provided through MMDB should add valuable additional knowledge for researchers working on the microbiome and related diseases when they use laboratory mouse. The database can be publicly accessible at http://leb.snu.ac.kr/mmdb/.

## Figures and Tables

**Table 1:** Summary statistics of the murine microbiome database (MMDB). OTU, operational taxonomic unit; SD, standard deviation.

	Counts
Total numbers of projects	14
Total numbers of samples	554
Mean OTUs/sample ± SD	657 ± 288
Mean valid reads/sample ± SD	61,280 ± 27,893
Number of sampling locations in gastrointestinal (GI) tract	7
Number of strains/genotypes	9
Number of vendors	10
Total number of phyla found in all samples	58
Total number of classes found in all samples	138
Total number of orders found in all samples	286
Total number of families found in all samples	585
Total number of genera found in all samples	1732
Total number of species found in all samples	4703

**Table 2:** Top five most frequently found bacterial species in the gastrointestinal tract of healthy laboratory mice. Values were calculated by considering samples with at least 1% relative abundance.

Sampling location	Name	Taxonomy	Proportions of samples (%)	Max (%)	Median (%)
**Stomach**	*Lactobacillus gasseri* group	*Firmicutes; Bacilli; Lactobacillales; Lactobacillaceae; Lactobacillus; Lactobacillus gasseri* group*;*	95.00	40.86	13.89
**(n = 20)**	*Lactobacillus reuteri* group	*Firmicutes; Bacilli; Lactobacillales; Lactobacillaceae; Lactobacillus; Lactobacillus reuteri* group*;*	95.00	55.55	11.64
	*Lactobacillus intestinalis*	*Firmicutes; Bacilli; Lactobacillales; Lactobacillaceae; Lactobacillus; Lactobacillus intestinalis;*	90.00	28.95	7.70
	PAC000185_s	*Bacteria; Proteobacteria; Alphaproteobacteria; Rhodospirillales; Rhodospirillaceae;* LARJ_g*;* PAC000185_s*;*	55.00	4.47	1.86
	PAC001472_s	*Bacteroidetes; Bacteroidia; Bacteroidales; Muribaculaceae;* PAC001472_g*;* PAC001472_s*;*	55.00	3.50	1.27
**Dudenum**	PAC001075_s	*Bacteroidetes; Bacteroidia; Bacteroidales; Muribaculaceae;* PAC000198_g*;* PAC001075_s*;*	93.75	6.30	2.07
**(n = 16)**	*Akkermansia muciniphila*	*Verrucomicrobia; Verrucomicrobiae; Verrucomicrobiales; Akkermansiaceae; Akkermansia; Akkermansia muciniphila;*	87.50	18.95	4.30
	*Lactobacillus gasseri* group	*Firmicutes; Bacilli; Lactobacillales; Lactobacillaceae; Lactobacillus; Lactobacillus gasseri* group*;*	81.25	36.58	5.20
	PAC001065_s group	*Bacteroidetes; Bacteroidia; Bacteroidales; Muribaculaceae;* PAC000186_g*;* PAC001065_s group*;*	81.25	7.48	4.06
	PAC001472_s	*Bacteroidetes; Bacteroidia; Bacteroidales; Muribaculaceae;* PAC001472_g*;* PAC001472_s*;*	81.25	9.17	3.75
**Jejunum**	PAC001065_s group	*Bacteroidetes; Bacteroidia; Bacteroidales; Muribaculaceae;* PAC000186_g*;* PAC001065_s group*;*	88.24	10.44	4.27
**(n = 34)**	PAC001075_s	*Bacteroidetes; Bacteroidia; Bacteroidales; Muribaculaceae;* PAC000198_g*;* PAC001075_s*;*	88.24	11.83	3.12
	*Lactobacillus gasseri* group	*Firmicutes; Bacilli; Lactobacillales; Lactobacillaceae; Lactobacillus; Lactobacillus gasseri* group*;*	85.29	39.67	6.85
	*Lactobacillus reuteri* group	*Firmicutes; Bacilli; Lactobacillales; Lactobacillaceae; Lactobacillus; Lactobacillus reuteri* group*;*	82.35	31.43	3.04
	*Lactobacillus intestinalis*	*Firmicutes; Bacilli; Lactobacillales; Lactobacillaceae; Lactobacillus; Lactobacillus intestinalis;*	79.41	37.06	3.55
**Ileum**	*Lactobacillus gasseri* group	*Firmicutes; Bacilli; Lactobacillales; Lactobacillaceae; Lactobacillus; Lactobacillus gasseri* group*;*	100.00	47.00	12.02
**(n = 20)**	*Lactobacillus reuteri* group	*Firmicutes; Bacilli; Lactobacillales; Lactobacillaceae; Lactobacillus; Lactobacillus reuteri* group*;*	95.00	39.17	5.93
	*Ileibacterium valens*	*Firmicutes; Erysipelotrichi; Erysipelotrichales; Erysipelotrichaceae; Ileibacterium; Ileibacterium valens;*	95.00	39.23	4.88
	PAC001472_s	*Bacteroidetes; Bacteroidia; Bacteroidales; Muribaculaceae;* PAC001472_g*;* PAC001472_s*;*	80.00	10.97	2.46
	PAC001065_s group	*Bacteroidetes; Bacteroidia; Bacteroidales; Muribaculaceae;* PAC000186_g*;* PAC001065_s group*;*	80.00	8.53	2.20
**Cecum**	PAC001188_s	*Firmicutes; Clostridia; Clostridiales; Ruminococcaceae; Oscillibacter;* PAC001188_s*;*	66.67	5.99	2.13
**(n = 96)**	KE159538_s	*Firmicutes; Clostridia; Clostridiales; Lachnospiraceae;* KE159538_g*;* KE159538_s*;*	45.83	20.38	4.38
	KE159714_s group	*Firmicutes; Clostridia; Clostridiales; Ruminococcaceae; Oscillibacter;* KE159714_s group*;*	41.67	16.71	2.03
	KE159628_s	*Bacteria; Firmicutes; Clostridia; Clostridiales; Lachnospiraceae;* KE159628_g*;* KE159628_s*;*	41.67	8.30	1.75
	PAC001080_s	*Tenericutes; Mollicutes; Acholeplasmatales; Acholeplasmataceae;* Acholeplasma_g2*;* PAC001080_s*;*	40.63	16.87	3.44
**Colon**	PAC001061_s	*Bacteroidetes; Bacteroidia; Bacteroidales; Rikenellaceae; Alistipes;* PAC001061_s*;*	70.00	10.61	3.47
**(n = 40)**	PAC001074_s	*Bacteroidetes; Bacteroidia; Bacteroidales; Muribaculaceae;* PAC001074_g*;* PAC001074_s*;*	67.50	5.83	2.11
	PAC001471_s	*Bacteroidetes; Bacteroidia; Bacteroidales; Rikenellaceae; Alistipes;* PAC001471_s*;*	52.50	12.00	5.04
	*Akkermansia muciniphila*	*Verrucomicrobia; Verrucomicrobiae; Verrucomicrobiales; Akkermansiaceae; Akkermansia; Akkermansia muciniphila;*	50.00	27.47	10.49
	PAC002478_s	*Bacteria; Proteobacteria; Deltaproteobacteria; Desulfovibrionales; Desulfovibrionaceae;* LT706945_g*;* PAC002478_s*;*	50.00	42.00	10.27
**Feces**	PAC001071_s	*Bacteroidetes; Bacteroidia; Bacteroidales; Muribaculaceae;* PAC001068_g*;* PAC001071_s*;*	77.13	58.21	4.01
**(n = 328)**	PAC001060_s	*Bacteroidetes; Bacteroidia; Bacteroidales; Rikenellaceae; Alistipes;* PAC001060_s*;*	55.79	52.82	6.78
	PAC001188_s	*Bacteria; Firmicutes; Clostridia; Clostridiales; Ruminococcaceae; Oscillibacter;* PAC001188_s*;*	49.70	18.59	2.18
	PAC001369_s group	*Bacteria; Firmicutes; Clostridia; Clostridiales; Ruminococcaceae; Oscillibacter;* PAC001369_s group*;*	39.63	12.95	1.99
	PAC001065_s group	*Bacteroidetes; Bacteroidia; Bacteroidales; Muribaculaceae;* PAC000186_g*;* PAC001065_s group*;*	37.50	13.89	2.23

## Supplementary Materials

The following are available online at https://www.mdpi.com/2076-2607/7/11/480/s1: Table S1: Wilcoxon rank-sum tests of differences in Chao1 index and Shannon index across different sampling locations, genotypes or vendors. Table S2: Pairwise ANOSIM results on the Bray–Curtis distance matrix across different sampling locations, genotypes or vendors with 999 permutations.
