# Supplementary material for: Introducing Murine Microbiome Database (MMDB): A Curated Database with Taxonomic Profiling of the Healthy Mouse Gastrointestinal Microbiome
Source: Microorganisms. 2019 Oct 23;7(11):480. doi: 10.3390/microorganisms7110480 (PMC6920994; doi:10.3390/microorganisms7110480)
Supplement: Supplementary file 1 [file microorganisms-07-00480-s001.pdf]

microorganisms

Supplementary Materials

Introducing Murine Microbiome Database (MMDB): A Curated Database with Taxonomic Profiling of the Healthy Mouse Gastrointestinal Microbiome

Junwon Yang [1,2], Jonghyun Park [1,2], Sein Park [1,2], Inwoo Baek [2,3] and Jongsik Chun [1,2,3,*]

[1] Interdisciplinary Program in Bioinformatics, Seoul National University, Seoul 00826, Republic of Korea; yjunwon18@gmail.com (J.Y.); warwarsn@naver.com (J.P.); psi103706@gmail.com (S.P.)
[2] Institute of Molecular Biology & Genetics, Seoul National University, Seoul 00826, Republic of Korea; iwbaek0828@gmail.com (I.B.)
[3] School of Biological Sciences, Seoul National University, Seoul 00826, Republic of Korea
* Correspondence: jchun@snu.ac.kr

Received: 11 September 2019; Accepted: 21 October 2019; Published: 23 October 2019

Table S1. Wilcoxon rank-sum tests of differences in Chao1 and Shannon indices across sampling locations, genotypes or vendors. Statistically significant comparisons ($P < 0.01$) are indicated in bold font.

	***P* value (Chao1)**	***P* value (Shannon)**
across sampling locations		
Cecum x Colon	0.001	0.002
Cecum x Duodenum	<0.001	0.787
Cecum x Feces	<0.001	<0.001
Cecum x Ileum	0.025	<0.001
Cecum x Jejunum	0.239	<0.001
Cecum x Stomach	0.001	<0.001
Colon x Duodenum	0.002	0.065
Colon x Feces	0.980	0.752
Colon x Ileum	<0.001	<0.001
Colon x Jejunum	0.001	<0.001
Colon x Stomach	0.127	0.029
Duodenum x Feces	<0.001	0.042
Duodenum x Ileum	<0.001	<0.001
Duodenum x Jejunum	<0.001	<0.001
Duodenum x Stomach	0.200	0.004
Feces x Ileum	<0.001	<0.001
Feces x Jejunum	<0.001	<0.001
Feces x Stomach	0.030	0.005
Ileum x Jejunum	0.160	0.058
Ileum x Stomach	<0.001	0.429
Jejunum x Stomach	0.001	0.951
across genotypes		
129S1/SvImJ x A/J	<0.001	0.993
129S1/SvImJ x BALB/c	<0.001	0.366
129S1/SvImJ x C57BL/6	<0.001	0.516
129S1/SvImJ x CAST/EiJ	0.174	0.536
129S1/SvImJ x NOD/LtJ	0.002	0.252
129S1/SvImJ x NZO/HILtJ	0.351	0.918
129S1/SvImJ x PWK/PhJ	0.232	0.694
129S1/SvImJ x WSB/EiJ	0.091	0.114
A/J x BALB/c	0.915	0.058
A/J x C57BL/6	0.009	0.029
A/J x CAST/EiJ	0.001	0.903
A/J x NOD/LtJ	0.001	0.349
A/J x NZO/HILtJ	<0.001	0.942
A/J x PWK/PhJ	<0.001	0.896
A/J x WSB/EiJ	<0.001	0.046
BALB/c x C57BL/6	0.008	0.761
BALB/c x CAST/EiJ	0.001	0.304
BALB/c x NOD/LtJ	<0.001	0.048
BALB/c x NZO/HILtJ	<0.001	0.350
BALB/c x PWK/PhJ	<0.001	0.353
BALB/c x WSB/EiJ	<0.001	0.455
C57BL/6 x CAST/EiJ	0.001	0.356
C57BL/6 x NOD/LtJ	<0.001	0.104
C57BL/6 x NZO/HILtJ	<0.001	0.476
C57BL/6 x PWK/PhJ	<0.001	0.509
C57BL/6 x WSB/EiJ	<0.001	0.506
CAST/EiJ x NOD/LtJ	0.094	0.340
CAST/EiJ x NZO/HILtJ	0.436	0.605
CAST/EiJ x PWK/PhJ	0.011	0.888
CAST/EiJ x WSB/EiJ	0.011	0.222
NOD/LtJ x NZO/HILtJ	0.019	0.113
NOD/LtJ x PWK/PhJ	<0.001	0.167
NOD/LtJ x WSB/EiJ	0.004	0.050
NZO/HILtJ x PWK/PhJ	0.015	0.815
NZO/HILtJ x WSB/EiJ	0.019	0.094
PWK/PhJ x WSB/EiJ	0.200	0.093
across vendors		
Animal Resource Centre x Charles River Laboratories	0.547	0.041
Animal Resource Centre x Harlan Envigo	0.463	0.363

Animal Resource Centre x Harlan Sprague Dawley	0.038	0.002
Animal Resource Centre x Jackson Laboratory	<0.001	0.026
Animal Resource Centre x Janvier	<0.001	0.002
Animal Resource Centre x National Laboratory Animal Center	0.583	0.720
Animal Resource Centre x Shanghai Laboratory Animal Center	0.006	0.001
Animal Resource Centre x Taconic farms	0.076	0.229
Animal Resource Centre x UAMS	0.169	0.014
Charles River Laboratories x Harlan Envigo	0.478	<0.001
Charles River Laboratories x Harlan Sprague Dawley	0.009	0.015
Charles River Laboratories x Jackson Laboratory	<0.001	<0.001
Charles River Laboratories x Janvier	<0.001	<0.001
Charles River Laboratories x National Laboratory Animal Center	0.914	0.151
Charles River Laboratories x Shanghai Laboratory Animal Center	0.012	0.140
Charles River Laboratories x Taconic farms	0.044	0.044
Charles River Laboratories x UAMS	0.355	<0.001
Harlan Envigo x Harlan Sprague Dawley	0.430	<0.001
Harlan Envigo x Jackson Laboratory	<0.001	0.071
Harlan Envigo x Janvier	0.001	0.084
Harlan Envigo x National Laboratory Animal Center	0.646	0.222
Harlan Envigo x Shanghai Laboratory Animal Center	0.229	<0.001
Harlan Envigo x Taconic farms	0.331	0.542
Harlan Envigo x UAMS	0.753	0.206
Harlan Sprague Dawley x Jackson Laboratory	<0.001	<0.001
Harlan Sprague Dawley x Janvier	<0.001	<0.001
Harlan Sprague Dawley x National Laboratory Animal Center	0.145	0.005
Harlan Sprague Dawley x Shanghai Laboratory Animal Center	0.173	0.930
Harlan Sprague Dawley x Taconic farms	0.038	0.019
Harlan Sprague Dawley x UAMS	0.122	<0.001
Jackson Laboratory x Janvier	0.093	0.203
Jackson Laboratory x National Laboratory Animal Center	<0.001	0.006
Jackson Laboratory x Shanghai Laboratory Animal Center	<0.001	<0.001
Jackson Laboratory x Taconic farms	0.395	0.445
Jackson Laboratory x UAMS	<0.001	0.583
Janvier x National Laboratory Animal Center	<0.001	<0.001
Janvier x Shanghai Laboratory Animal Center	<0.001	<0.001
Janvier x Taconic farms	0.623	0.701
Janvier x UAMS	<0.001	0.478
National Laboratory Animal Center x Shanghai Laboratory Animal Center	0.033	0.017
National Laboratory Animal Center x Taconic farms	0.100	0.067
National Laboratory Animal Center x UAMS	0.592	0.003
Shanghai Laboratory Animal Center x Taconic farms	0.009	0.009
Shanghai Laboratory Animal Center x UAMS	0.049	<0.001
Taconic farms x UAMS	0.017	0.424

Table S2. Pairwise ANOSIM results on the Bray-Curtis distance matrix across different sampling locations, genotypes or vendors with 999 permutations.

	Sample size	*R* value	*P* value
across sampling locations			
Cecum x Colon	136	0.390	0.001
Cecum x Duodenum	112	0.325	0.001
Cecum x Feces	424	0.288	0.001
Cecum x Ileum	116	0.622	0.001
Cecum x Jejunum	130	0.601	0.001
Cecum x Stomach	116	0.466	0.001
Colon x Duodenum	56	0.200	0.002
Colon x Feces	368	0.338	0.001
Colon x Ileum	60	0.501	0.001
Colon x Jejunum	74	0.505	0.001
Colon x Stomach	60	0.417	0.001
Duodenum x Feces	344	0.264	0.001
Duodenum x Ileum	36	0.343	0.001
Duodenum x Jejunum	50	0.074	0.087
Duodenum x Stomach	36	0.427	0.001
Feces x Ileum	348	0.689	0.001
Feces x Jejunum	362	0.592	0.001
Feces x Stomach	348	0.536	0.001
Ileum x Jejunum	54	0.068	0.066

Ileum x Stomach	40	0.324	0.001
Jejunum x Stomach	54	0.417	0.001
across genotypes			
129S1/SvImJ x A/J	73	0.507	0.001
129S1/SvImJ x BALB/c	64	0.564	0.001
129S1/SvImJ x C57BL/6	387	0.531	0.001
129S1/SvImJ x CAST/EiJ	16	0.206	0.014
129S1/SvImJ x NOD/LtJ	16	0.522	0.001
129S1/SvImJ x NZO/HILtJ	16	0.484	0.001
129S1/SvImJ x PWK/PhJ	15	0.755	0.001
129S1/SvImJ x WSB/EiJ	16	0.573	0.001
A/J x BALB/c	123	0.155	0.001
A/J x C57BL/6	446	0.157	0.001
A/J x CAST/EiJ	75	0.544	0.001
A/J x NOD/LtJ	75	0.540	0.001
A/J x NZO/HILtJ	75	0.454	0.001
A/J x PWK/PhJ	74	0.363	0.001
A/J x WSB/EiJ	75	0.566	0.001
BALB/c x C57BL/6	437	0.111	0.003
BALB/c x CAST/EiJ	66	0.626	0.001
BALB/c x NOD/LtJ	66	0.542	0.001
BALB/c x NZO/HILtJ	66	0.545	0.001
BALB/c x PWK/PhJ	65	0.474	0.001
BALB/c x WSB/EiJ	66	0.696	0.001
C57BL/6 x CAST/EiJ	389	0.572	0.001
C57BL/6 x NOD/LtJ	389	0.418	0.001
C57BL/6 x NZO/HILtJ	389	0.514	0.001
C57BL/6 x PWK/PhJ	388	0.573	0.001
C57BL/6 x WSB/EiJ	389	0.611	0.001
CAST/EiJ x NOD/LtJ	18	0.607	0.001
CAST/EiJ x NZO/HILtJ	18	0.418	0.001
CAST/EiJ x PWK/PhJ	17	0.503	0.001
CAST/EiJ x WSB/EiJ	18	0.358	0.001
NOD/LtJ x NZO/HILtJ	18	0.675	0.001
NOD/LtJ x PWK/PhJ	17	0.798	0.001
NOD/LtJ x WSB/EiJ	18	0.753	0.001
NZO/HILtJ x PWK/PhJ	17	0.766	0.001
NZO/HILtJ x WSB/EiJ	18	0.286	0.001
PWK/PhJ x WSB/EiJ	17	0.700	0.001
across vendors			
Animal Resource Centre x Charles River Laboratories	79	0.495	0.001
Animal Resource Centre x Harlan Envigo	154	0.810	0.001
Animal Resource Centre x Harlan Sprague Dawley	104	0.796	0.001
Animal Resource Centre x Jackson Laboratory	180	0.578	0.001
Animal Resource Centre x Janvier	33	0.999	0.001
Animal Resource Centre x National Laboratory Animal Center	27	0.915	0.001
Animal Resource Centre x Shanghai Laboratory Animal Center	33	0.912	0.001
Animal Resource Centre x Taconic farms	15	0.999	0.018
Animal Resource Centre x UAMS	33	1.000	0.001
Charles River Laboratories x Harlan Envigo	207	0.635	0.001
Charles River Laboratories x Harlan Sprague Dawley	157	0.609	0.001
Charles River Laboratories x Jackson Laboratory	233	0.566	0.001
Charles River Laboratories x Janvier	86	0.604	0.001
Charles River Laboratories x National Laboratory Animal Center	80	0.668	0.001
Charles River Laboratories x Shanghai Laboratory Animal Center	86	0.478	0.001
Charles River Laboratories x Taconic farms	68	0.866	0.001
Charles River Laboratories x UAMS	86	0.747	0.001
Harlan Envigo x Harlan Sprague Dawley	232	0.801	0.001
Harlan Envigo x Jackson Laboratory	308	0.924	0.001
Harlan Envigo x Janvier	161	0.905	0.001
Harlan Envigo x National Laboratory Animal Center	155	0.861	0.001
Harlan Envigo x Shanghai Laboratory Animal Center	161	0.714	0.001
Harlan Envigo x Taconic farms	143	0.965	0.001
Harlan Envigo x UAMS	161	0.937	0.001
Harlan Sprague Dawley x Jackson Laboratory	258	0.542	0.001
Harlan Sprague Dawley x Janvier	111	0.943	0.001
Harlan Sprague Dawley x National Laboratory Animal Center	105	0.936	0.001
Harlan Sprague Dawley x Shanghai Laboratory Animal Center	111	0.683	0.001
Harlan Sprague Dawley x Taconic farms	93	0.987	0.001

Harlan Sprague Dawley x UAMS	111	0.976	0.001
Jackson Laboratory x Janvier	187	0.845	0.001
Jackson Laboratory x National Laboratory Animal Center	181	0.757	0.001
Jackson Laboratory x Shanghai Laboratory Animal Center	187	0.567	0.001
Jackson Laboratory x Taconic farms	169	0.714	0.002
Jackson Laboratory x UAMS	187	0.142	0.002
Janvier x National Laboratory Animal Center	34	0.985	0.001
Janvier x Shanghai Laboratory Animal Center	40	0.999	0.001
Janvier x Taconic farms	22	0.999	0.004
Janvier x UAMS	40	0.999	0.001
National Laboratory Animal Center x Shanghai Laboratory Animal Center	34	0.932	0.001
National Laboratory Animal Center x Taconic farms	16	1.000	0.009
National Laboratory Animal Center x UAMS	34	0.998	0.001
Shanghai Laboratory Animal Center x Taconic farms	22	0.999	0.006
Shanghai Laboratory Animal Center x UAMS	40	1.000	0.001
Taconic farms x UAMS	22	0.979	0.005
